# Age-Related Cognitive and Volumetric Changes in the Brain of African Grasscutter (*Thryonomys swinderianus* (Temminck, 1827))

**DOI:** 10.1155/vmi/3349981

**Published:** 2024-12-13

**Authors:** Hounakey M. Afanvi, Maman You Esperance Broalet, Ouattara Niemtiah, Yaovi James, Brahima Doukoure, Neme Antoine Tako, Kossi Metowogo, Kwashi Eklu-Gadegbeku, Kodjo Aklikokou

**Affiliations:** ^1^Laboratory of Biology and Health, Neuroscience Unit, Felix Houphouët-Boigny University of Abidjan, BP V34, Abidjan 01, Ivory Coast; ^2^Anatomy Laboratory, Medical Science Training and Research Unit, Alassane Ouattara University of Bouake, BP V 18 01, Bouake, Ivory Coast; ^3^Anatomy Laboratory, Faculty of Health Sciences, University of Lome, 01BP: 1515, Lome, Togo; ^4^Pathological Anatomy and Cytology Laboratory, Training and Research Unit-Medical Sciences, Felix Houphouët-Boigny University of Abidjan, BP V34, Abidjan 01, Ivory Coast; ^5^Physiology-Pharmacology Laboratory, Physiopathology Bioactive Substances and Safety Research Unit, University of Lome, 01BP: 1515, Lome, Togo

**Keywords:** African grasscutter, aging, brain, cognition, volume, weight

## Abstract

The African grasscutter (AGC) (*Thryonomys swinderianus*) is the second largest rodent in sub-Saharan Africa. It is bred for its organoleptic and culinary properties but also serves as a research model. The aim of this study was to investigate the relationship between age-related changes in brain weight, brain volume, and spatial and nonspatial memory performance in the AGC. A total of forty-two (42) captive-derived AGCs were divided into seven age groups: 6 neonates (6 days), 6 juveniles (1 month), 6 pubertals (3 months), 6 subadults (8 months), 6 young adults (2 years), 6 middle adults (4 years), and 6 old adults (5 years). The subjects were given a series of tests to assess their spatial memory (location test) and their nonspatial memory (object recognition test). Brain samples were then collected using basic neuroanatomical techniques. The weight and volume of the brain samples were determined and their encephalization quotient (EQ) was also calculated. The results showed that spatial and nonspatial memory in AGC develops into adulthood and then declines with age. Mean brain weight increased from neonates to mid-adulthood (5.20 ± 0.31 g–11.76 ± 0.23 g) and decreased in older AGC (11.75 ± 0.22 g). In contrast, the mean weight of the olfactory bulb (OB) increased from neonates to young adults (0.28 ± 0.02 g–0.80 ± 0.03 g) and the cerebellum increased from neonates to older (0.59 ± 0 0.01 g–1.86 ± 0.06 g). Finally, the EQ decreased with age (0.73 ± 0.05–0.29 ± 0.01). Mean brain volume increased with age from neonates to young adult (5 mL–11.25 mL). Conversely, the mean volume of the OB and cerebellum increases from neonates to older age (0.33 ± 0.03 mL–0.95 ± 0.04 mL). This study shows that spatial and nonspatial memory declines during the aging process in AGC. Neonates and juveniles have higher cognitive abilities than adults and older AGC. The weight of the brain, OB, and cerebellum increases from neonates to adult and decreases slightly from middle to old adults. However, the volume of the OB and cerebellum increases with age.

## 1. Introduction

In recent decades, life expectancy has increased in humans [1], and mortality among the elderly has decreased [2]. In mammals, aging is characterized by a gradual decline in physiological functions and responses involving organs and tissues. For example, a variety of changes occur with age, including brain atrophy, oxidative stress, and reduced antioxidant defenses, which contribute to impaired learning, memory, and physical activity [3]. In the elderly population, cognitive impairment is becoming a major health problem and requires considerable efforts to determine their neural basis, etiology, and treatment [4]. To achieve this goal, there is an urgent need for comprehensive information on functional and structural changes in the brain across the lifespan [5]. Experimental studies in animal models are important to investigate changes in cognition and cerebral morphometry.

Animal models offer important practical advantages over human research. Most importantly, from a gerontological perspective, the lifespan of laboratory animals is typically much shorter than that of humans. Secondly, the ethical considerations of human research do not permit the types of invasive analyses and treatments that can be carried out in animal models. Finally, the control of genetic and environmental factors in animal research is seen as an advantage over human studies, which can be affected by wide variations [6]. Rodents are the largest mammalian order with approximately 2277 species [7] and are most commonly used in animal experimentation [8].

The African grasscutter (AGC) is a member of the suborder Hystricomorpha and the family of Thryonomyidae. The AGC is a large rodent, the second largest rodent in sub-Saharan Africa after the northern crested porcupine [9]. AGCs are prevalent in the West African subregion from Senegal to parts of the Cape Province including Ghana, Nigeria, Togo, Benin, and Côte d'Ivoire [10]. This indigenous African rodent possesses uncommon phenotypes, life histories, and a brain that has attracted and fanned the curiosity of African scientists [11, 12]. Its average lifespan in captivity varies from seven (7) to nine (9) years. It can be up to 12 years depending on the health care given to the animals during their lifetime [13]. Studies have shown that AGC can be tamed and even used for laboratory animals [14]. The development of indigenous research animals such as AGC should be encouraged. Because rodent rearing involves all stages of development, it was necessary to understand how the brain changes during normal aging before the boundary between normal and pathological states can be fully understood.

Although numerous studies on cognition and structural changes in the brain during aging have been conducted in monkeys, mice, dogs, cows [15], pigs and horses [16], and mouse lemurs [17], to the best of our knowledge, little scientific information has been provided reported on the aging AGC (*Thryonomys swinderianus*) in captivity. Given these observations, we undertook a study of the AGC in close captivity. The aim of the current study is to investigate the relationship between age-related changes in spatial and nonspatial memory performance, brain weight, and brain volume. The results of this study will contribute significantly to the growing neurobiological database on the AGC.

## 2. Materials and Methods

### 2.1. Ethical Approval

The experiments in AGC were performed according to the guidelines for the use of research animals in the Laboratory of Animal Physiology at the University of Lome (001/2012/CB-MSDS-UL) in accordance with the internationally accepted principles for laboratory animal use and care, as set out in Directive 86/609/EEC and the National Research Council's Guide for the Care and Use of Laboratory Animals, 8^th^ Edition, USA

### 2.2. Experimental Animals

This experimental study was conducted on 42 male AGCs in captivity, including 6 neonates aged 6 days, 6 juveniles aged 30 days (1 month), 6 pubertals aged 90 days (3 months), 6 subadults aged 240 days (8 months), 6 young adults aged 720 days (2 years), 6 middle adults aged 1400 days (4 years), and 6 old adults aged 1800 days (5 years). These animals were maintained at a temperature of 25 ± 2°C, humidity of 50%, and natural photoperiod. Food and water were provided daily *ad libitum*. The foods consumed consisted of maize stalks, maize seeds, green papaya, cassava pods, cassava and tuber peels and waste, cereal bran (maize, sorghum, millet, rice, and wheat), groundnut and palm kernel cakes, table salt, cassava stalks, etc. The food was given before supplementation to prevent digestive problems, most commonly abdominal bloating. The veterinarians did not find any clinical signs of diseases, apparent deformities, or behavioral disorders in these animals.

### 2.3. Memory Test

The apparatus used for this test is an open field. It consisted of a square arena surrounded by a wall (height = 1.8 m, length = 2 m, width = 2 m) with a white painted background.

#### 2.3.1. Object Localization Test (OLT)

To assess spatial memory, the object location test was used according to the method of Denninger, Smith, and Kirby [18] and Vogel-Ciernia and Wood [19]. The test was administered in four (4) steps: familiarization and acclimatization (first step), habituation (second step), training (third step), and testing (fourth step). The AGCs were frequently manipulated by the experimenter to avoid being stressed by his presence during the first step. In the second step, the AGCs were returned to the experimental room for acclimatization and a free exploration period of ten (10) minutes in the open field. Each animal received three (3) trials per day. Before each trial, the arena was always swept and cleaned with 70% ethanol to remove odors and other materials. Fifteen minutes were allowed for the ethanol to evaporate completely. After the three trials, the AGCs were returned to their original enclosures. Twenty-four (24) hours after the habituation phase, the training phase followed. In parallel quadrants of the arena (NE corner and NW corner), heavy objects of different shapes and colors were placed. Each AGC was removed from its cage and placed in the center of the arena, equidistant from the 2 identical objects. Each AGC freely explored the arena and the objects for 10 min. At the end of the test, the AGC was removed and placed in the holding cage for one hour. Finally, in the last step, the object in the NW corner was moved to the SW quadrant. Each AGC was then freed to explore the arena and the objects for 10 min.

#### 2.3.2. New Object Recognition Test (NORT)

The NORT or object recognition test (ORT) was originally described by Ennaceur and Delacour [20]. The ORT is identical to the OLT until the fourth step, where the moved object (MO) is replaced by a new object (NO). The object that was moved during the OLT (NW quadrant) was replaced by a NO. Each animal was removed from its cage and placed in the center of the arena, equidistant from the 2 objects. Each AGC was allowed to freely explore the arena and the objects for 10 min. At the end of each trial, the AGCs were returned to their original cage.

#### 2.3.3. Variables Measured

The total exploration time of the NO or MO and the familiar object (FO) was measured for each session to calculate the total percentage of investigation time and the discrimination index.

(1) $$\begin{array}{c} \text{Total percentage of investigation time}=\frac{\text{total time investigation of NO or MO}}{\text{total time investigation of NO or MO}+\text{total time investigation of FO}}*100. \end{array}$$

A value greater than 50% indicates a very long investigation time of the MO or NO.

(2) $$\begin{array}{c} \text{Discrimination index}\left(\text{DI}\right)=\frac{\text{Investigation time of NO or MO}-\text{Investigation time of FO}}{\text{Investigation time ofNO or MO}+\text{Investigation time of FO}}. \end{array}$$

DI < 0: AGCs explore the FO/stationary object more than the NO/MO.

DI > 0: AGCs explore the NO/MO more than the FO/stationary object.

### 2.4. Brain Weight and Volume Measurements

The next day, all animals were anesthetized with acepromazine and ketamine at doses of 0.5 mg/kg body weight and 0.7 mg/kg, respectively. Because AGCs' blood clots very quickly, they were also injected with standard heparin (5000 IU) to prevent blood clotting during anatomical dissection. They were measured using a PAFEX® 25 kg mechanical dial scale. The scale had a 17 cm diameter and displayed measurements in 100 g increments, with a precision of 0.01 g. The animals were then sacrificed and the brains were extracted. Whole brain (WB), OBs, and cerebellum weights were measured using a Mettler P 1261 balance (Mettler AG, Greifensee, Switzerland) with a sensitivity of 0.01 g. The relative brain weight was calculated by dividing the absolute brain weight by the body weight, expressed as a percentage [21]. Volumes for the WB, OB, and cerebellum were determined using the water displacement method [22].

### 2.5. EQ

The EQ was calculated from the weight of the brain and body using the Deaner et al. [23] equation: EQ = *M*brain/0.12∗*M*body^0.67^.

EQ = encephalization quotient.

Mbrain = absolute brain weight [g].

Mbody = body weight [g].

The interpretation of EQ was based on that of Shoshani, Kupsky, and Marchant [24].

### 2.6. Statistical Analysis

All data are presented as the mean and standard error of the mean (mean ± SEM). To assess the statistical difference between mean values, a one-way analysis of variance (ANOVA) was conducted, followed by comparisons using Tukey's post hoc test. Statistical analysis was conducted with Excel 2019 (Microsoft Office 2019) and the statistical package (GraphPad Prism 8.0, San Diego, California, USA). A *p* value of *<* 0.05 was considered to be statistically significant. The weight and volume were analyzed statistically for correlation with age.

## 3. Results

### 3.1. Memory in Aging AGC

**Figure 1:**
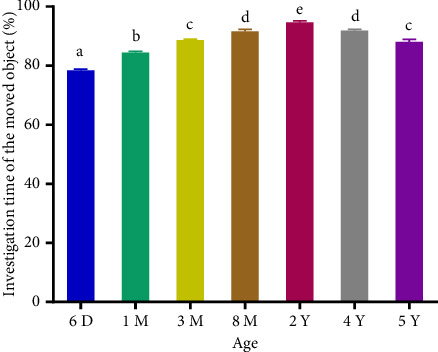
Investigation time of the moved object in aging AGC. 6D: 6 days, 1M: 1 month, 3M: 3 months, 8M: 8 months, 2Y: years, 4Y: 4 years, and 5Y: 5 years. Averages with different letters above the bars show a significant difference (*p* < 0.05) between ages. Those with the same letters show no significant difference (*p* > 0.05) between ages. The percentage of investigation time increased between 6 days and 2 years followed by a decrease between 2 years and 5 years.

**Figure 2:**
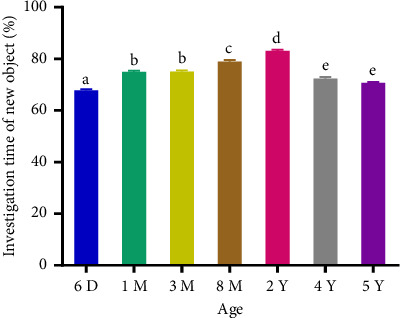
Investigation time of the new object in aging AGC. 6D: 6 days, 1M: 1 month, 3M: 3 months, 8M: 8 months, 2Y: years, 4Y: 4 years, and 5Y: 5 years. Averages with different letters above the bars show a significant difference (*p* < 0.05) between ages. Those with the same letters show no significant difference (*p* > 0.05) between ages. The percentage of investigation time increased between 6 days and 2 years followed by a decrease between 2 years and 5 years.

**Figure 3:**
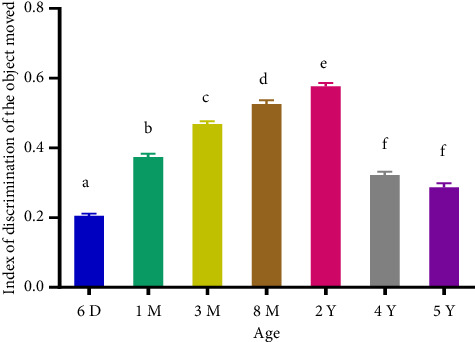
Moved object discrimination index in aging AGC. 6D: 6 days, 1M: 1 month, 3M: 3 months, 8M: 8 months, 2Y: years, 4Y: 4 years, and 5Y: 5 years. Averages with different letters above the bars show a significant difference (*p* < 0.05) between ages. Those with the same letters show no significant difference (*p* > 0.05) between ages. The index of discrimination of the moved object increased between 6 days and 2 years followed by a decrease between 2 years and 5 years.

**Figure 4:**
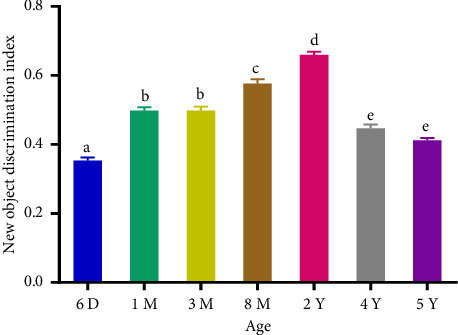
New object discrimination index in aging AGC. 6D: 6 days, 1M: 1 month, 3M: 3 months, 8M: 8 months, 2Y: years, 4Y: 4 years, and 5Y: 5 years. Averages with different letters above the bars show a significant difference (*p* < 0.05) between ages. Those with the same letters show no significant difference (*p* > 0.05) between ages. The discrimination index increased between 6 days and 2 years followed by a decrease between 2 years and 5 years.

The total percentage of investigation time increased significantly (*p* < 0.0001) from AGC aged 6 days to 2 years and decreased from 2 to 5 years (Figures 1 and 2). Adults spent more time exploring the displaced and the NO compared to subadults, young, newborns, and the old adults. The positive and significant increase (*p* < 0.001) in the mean DI between 6 days and 2 years of birth indicates that the investigation of the displaced and novel object increases until adulthood. Its positive and significant decrease from 2 to 5 years of age indicates that old adults spent less time around the displaced and novel object compared to adults (Figures 3 and 4). The spatial and nonspatial memory of the AGC evolves into adulthood and then decreases in older AGC.

### 3.2. Weight

The mean body weight of the AGC increased significantly (*p* < 0.001) from 6 days to 5 years (451.3 ± 19.5 g to 5967.7 ± 21.33 g) (Table 1). The brain weight also increases from 5.20 ± 0.31 g to 11.76 ± 0.41 g between 6 days of birth and 4 years of age (Table 1). It decreased significantly to 11.71 ± 0.22 g at 5 years of age. But, the relative weight of the brain decreased with age from 1.16 ± 0.09% to 0.20 ± 0.00% (Table 1). There were no significant differences between the age groups, except for young adults and old adults. The percent change in brain weight showed a decrease from 6 days to 5 years. Growth was strong and positive at one month (58.08%), but weak and negative after 5 years (−0.09%) (Table 1). The mean weight of the OB increased from 6 days of age to 2 years of age (0.28 ± 0.02 g to 0.80 ± 0.03 g) (Table 1). The statistical analysis was significant (*p* < 0.05) between age groups except from neonates to young, juveniles to subadults, and young adults to old age. The cerebellum weight also increased from 0.59 ± 0.01 g to 1.86 ± 0.06 g between 6 days of age and 5 years of age, with stabilization from 2 years of age (Table 1). The difference between ages was significant (*p* < 0.05), except between neonates and juveniles, and between young adults and old adults.

#### 3.2.1. EQ

The mean EQ decreased with age from 0.73 ± 0.05 to 0.29 ± 0.01 (Table 1). The difference was significant between the age groups (*p* < 0.05), except between neonates and juveniles, and also between young adults and old adults. AGC EQ scores less than 1 reflect lower than average intelligence, which declines with age.

### 3.3. Volume

The mean brain volume increased from 5 ± 0.29 mL to 11.25 ± 0.21 mL between 6 days of age and 2 years of age (Table 2). It then remained constant between the ages of 2 and 4 years. However, it decreases significantly from 11.25 ± 0.21 mL to 11.17 ± 0.28 mL between 4 and 5 years. The statistical analysis was significant (*p* < 0.0001) between the age groups, except for juveniles and old adults.

The mean volume of the OB increased from 0.33 ± 0.03 mL to 0.95 ± 0.04 mL between 6 days of age and 5 years of age (Table 2). The statistical analysis was significant (*p* < 0.05) across the various age groups, except between neonates and young juveniles, as well as those between subadults and the old adults groups.

The mean volume of the cerebellum increased from 0.85 ± 0.06 mL to 3.40 ± 0.06 mL between 6 days of birth and 5 years of age, with stabilization from 2 years of age (Table 2). The statistical analysis was significant between ages except for middle adults and older adults.

## 4. Discussion

### 4.1. Memory in Aging AGC

The OLT used in this work is used to measure hippocampus-dependent spatial memory [25]. Our data are in agreement with the work done in humans where it has been shown a loss of spatial memory abilities with age [26]. The same results were found by Wyss et al. [27] who used the water maze task to show that learning and spatial memory in Sprague Dawley rats, spontaneously hypertensive rats, and Wistar Kyoto rats decreased with age. Also, Hendrickx et al. [28] used the Morris water maze and the object location test to show that there is a decrease in spatial memory in C57BL/6J mice from 6 months of age. Spatial memory impairment is associated with impairment of hippocampal cells, circuits, and plasticity [29]. CA1 pyramidal cells respond gradually to gradual changes in the environment [30].

The present work also allows us to measure the nonspatial memory in the AGC. Our data show that nonspatial memory of the AGC evolves into adulthood and decreases in the elderly; this is in agreement with the numerous studies that postulate that episodic memory gradually deteriorates from the age of 60 in humans [31]. This decline may also begin earlier in adulthood [32]. NORTs in mice have shown that long-term memory is impaired with age when the inter-trial interval time is greater than 10 min [33].

In mammals, spatial memory and nonspatial memory depend on the hippocampus and associated structures at the medial temporal lobe [34]. Several mechanisms have been suggested to explain the age-related decline in recognition memory, including morphological changes in the hippocampus, alteration of hippocampal location cell properties [35], or reduced synaptic plasticity [36]. It is also well established that age-related functional changes in the perirhinal cortex induce a reduction in the ability of animals to disambiguate between NOs in familiars and contribute to impaired object recognition [37].

### 4.2. Weight

Data from the present study revealed that the increase in the body weight from PND6 to PND1800 was statistically significant. This increase was also noted by Ibe et al. [38, 39]. But in our study, the body weights found in the 6-day- and 720-day-old were high compared to those found by Ibe et al. [38, 39]. Nadon [40] and Turturro et al. [41] showed that *ad libitum* fed mice continue to grow and gain weight over a considerable part of their lifespan. After an initial fast increase covering about 4 weeks, growth continues but at a slower pace until weight peaks at 15–20 months. At advanced age (> 24 months), body weight decreases. But, Altun et al. [42] showed that beyond two years, the animals maintain their weight or disclose a modest decrease; this loss in whole body mass accelerates in old age. Study in *Mus musculus* showed that aging is also marked by increased body weight, which was observed from the ages of 24–48 weeks [43]. Body weight growth in rats has two stages: (1) development to maturity in which growth rate is high, and all parts of the body grow, (2) post maturity growth in which the growth rate is lower than the previous stage [44]. Chinedu et al. [45] showed that increases in body weight in humans are clearly found from age 13–19 years up to 40–59 years. After the age of 40–59 years, the rate of weight increase showed a static trend. Importantly, the decrease in gross body mass in senescence is associated with an apparently involuntary change in body mass composition [42]. Weight gain and loss are determined by a complex interplay of health, lifestyle, and genetic factors [46]. Physiological changes in body composition that influence the loss of body weight with age include decrease in cell mass through cell death and impaired function of surviving cells [47], decrease of body fat and body water [48], and changes in internal organs, bone mass, skeletal muscles, and lean body mass in elderly subjects [47, 49].

The present study revealed the increase in brain weight from 6 days to 4 years, followed by a slight decrease at 5 years. Broalet et al. [12] found 8.4 ± 1.78 g; 10.51 ± 2 g; and 10.78 ± 3.71 g, respectively, in juveniles (1 month); pubescents (3 months), and young adults (7–8 months). These results show a growth in brain weight with age, but the value found in young adults (11.71 ± 0.37 g) is higher than that found by Broalet et al. [12]. Ojo et al. [39] also found different values in neonates (4.19 ± 0.08 g), juveniles (6.77 ± 0.04 g), and older adults (12.22 ± 0.23 g). Olude, Mustapha, and Olopade [50] found in the African giant rat (*Cricetomys gambianus*, Waterhouse) values that also increase with age (neonate = 0.62 ± 0.08 g; juvenile = 4.64 ± 0.17 g; and adult = 5.60 ± 0.06 g). However, the average brain weight of the adult AGC was slightly higher than that of the African giant rat [50], adult guinea pig (4 g), adult large cane rat (9.80 ± 0.50 g for males and 10.27 ± 0.45 g for females, [51]), and squirrel (7.6 g, [52]), but lower than porcupine (25 g) [51]. Piao, Liu, and Xie [53] showed that age-related growth in brain weight in Sprague Dawley rats was not significant. Although many report neuronal loss in the cerebral cortex, striatum, and hippocampus, as well as an increase in TUNNEL-positive cells in the parietal lobe, corpus callosum, medulla oblongata, and cerebellum, neuronal loss can be compensated for by an increase in neuroglial cells, which leads to an increase in brain weight with increasing age [54, 55]. Researchers have found that after the third or sixth decade of life, the weight of the human brain decreases [56]. A 36% decrease in the solid portion of the brain is seen, which may indicate a loss of solid brain material. In the brain, there was a slight rise in the concentration of water, but a decrease in the concentration of a number of lipids. A large portion of the solid brain material would therefore be lost as people aged, and some would be replaced by water [57]. There has been an increase or a relatively constant brain weight observed in other studies. In line with our research, Penzes, Izsak, and Beregi [58] revealed that in Sprague Dawley rats, there was an increase in brain weight (normalized to body weight). Because the ratio of brain weight to body weight varies among species, normalization makes it impossible to compare data from different species [59]. The increased brain weight apparently reflects increases in brain ventricular volume in the study of Chen, Tung, and Chang [60]. But, the differences in brain weight could be explained by the influence of certain environmental factors such as sensory stimuli [61], which tend to increase brain weight, and domestication, which tends to lighten the brain, and tension-related factors can influence these values [62].

In this study, the absolute OB weights of juveniles and subadults were 0.31 ± 0.02 g and 0.56 ± 0.04 g, respectively. These values differ slightly from those found by Byanet, Onyeanusi, and Ibrahim [51] in male juveniles (0.57 ± 0.05 g) and subadult females (0.43 ± 0.10 g). This was indicative of increased neuronal and neuroglial cell size.

The weight of the cerebellum increases from newborn to old adult. Ojo et al. [39] also found an increase in cerebellar weight from newborn (P3) to adult (P450). The weight gain is explained by cerebellar neurogenesis and the very rapid increase in neuron size in newborns [39]. In humans, cerebellar weight decreases with age [63].

EQ results suggest higher memory and cognition in neonates and juveniles compared to adults and aged AGC. The EQ of adult AGC recorded in the present study is similar to the values of 0.49 and 0.40, respectively, reported by Ojo et al. [39] and Byanet and Dzenda [64] but is above 0.19 reported for adult African giant pouched rat [65]. Our values of the EQ of neonates and juvenile AGC are lower than those found by Ojo et al. [39]. These differences in values could be explained by factors such as gender, body mass, neuron density, cortical thickness, number of neurons, brain folding, and the development of the cerebral cortex [66].

### 4.3. Volume

The present study revealed an increase in brain volume. Ojo et al. [39] found the same increase. In humans, the brain volume increases during the first years of life and generally decreases during the last years [67]. Human brain volume has been found to slightly decrease with age, while ventricular volume has increased, according to MRI studies [68]. The rate of change in brain volume is negatively correlated with age, according to research by Takao, Hayashi, and Ohtomo [69]. Brain volume growth can be explained by two mechanisms: cell proliferation, dendritic branching and synaptogenesis, and myelination [70]. The brain undergoes atrophy during the aging process, and the shrinkage has been well characterized in many studies [71]. In a cross-sectional study on nondemented adults, a significant decline in WB volume was detected in early adulthood and continued into old age, with distinct patterns for gray and white matter loss [72]. Longitudinal studies have also reported annual gross brain volume decreases on the order of 0.2%–0.5% after the age of 47 [67]. During normal aging, discrete atrophy of the cortex may be due, in part, to cell body atrophy and decreased neuron arborization [73]. This atrophy can be explained by cell death and the elimination of dendrites, axons, and therefore synapses [74].

The AGC, like other rodents, has a strong sense of smell [14]. Data from the present study revealed that the volume of the OB increased from neonates to old age. The results of this study differ from those of Lessard-Beaudoin et al. [75] in C57BL/6 mice. However, they are consistent with two other studies in Sprague Dawley rats in which the volume of the OB increases with age [76]. The authors assume that the increase occurs uniformly throughout the bulb, as no significant change in the proportions of the different layers was observed. Furthermore, few apoptotic cells were observed, a sign that the OB circuitry remains stable with age [76]. Because many changes occur in the brain with age, it is possible that the difference between our results and theirs is due to a change in the composition of the OB that alters the relationship between volume and weight [77]. Mirich et al. [76] explained that the volume of the OB during aging depends on both rodent strains and species. Kaas [78] attributed changes in OB volume to the number of neurons, the size of such neurons, and/or their connectivity. Haehner et al. [79] showed OB volume to increase with olfaction ability. In humans, OB volume in healthy adults appears to decrease with age parallel with olfactory function [80]. As indicated by correlational analyses in the study of Hummel et al. [81], OB volumes of children increase in parallel to smell function with advancing age. Previous empirical studies have also shown that thresholds in children and adolescents are either lower than those of adults [82] or the same as in adults [85]. These differences over the lifespan may, however, depend not only on changes in olfactory sensitivity but also on cognitive abilities [83].

The cerebellum has been implicated in balance, timing, sensorimotor adaptation, associative learning (eye-blink conditioning), working memory, and attention [84]. Data from the present study revealed that the volume of the cerebellum increased from neonates to old age. On the other hand, Hamezah et al. [85] showed that the cerebellum volume was not affected by age in male Sprague Dawley rats. In humans, the cerebellar morphology has been investigated in both pediatric and aging populations. Some studies reported that cerebellar volume remains relatively stable with aging [86], whereas others found strong effects of age on cerebellar atrophy [87]. A histological study showed that the loss of cerebellar volume was mainly due to white matter loss [88].

## 5. Conclusion

This study reveals that spatial and nonspatial memory decreases in aging AGC. The volume of the brain, OB, and cerebellum increases with age, and the weight of the brain increases with age but begins to stabilize at 4 years of age. The weight of the OB begins to stabilize at the age of 4 years after rapid growth, but the weight of the cerebellum increases with age. AGC newborns and juveniles have a higher cognitive capacity than adults and the elderly. Therefore, juveniles should be preferred in physiological studies of memory and cognition.

## Figures and Tables

**Table 1:** Weight parameters of African grasscutter brain.

Weight		Number of samples (*n* = 42)						
		6 days	1 month	3 months	8 months	2 years	4 years	5 years
Average ± SEM	BW (g)	451.33 ± 19.25^**a**^	878 ± 17.31^**b**^	1464 ± 19.06^**c**^	2681.17 ± 18.43^**d**^	4561.83 ± 18.02^**e**^	5111.33 ± 21.25^**f**^	5967.17 ± 21.33^**g**^
	BRW (g)	5.20 ± 0.31^**a**^	8.22 ± 0.31^**b**^	10.23 ± 0.37^**c**^	11.71 ± 0.37^**d**^	11.76 ± 0.41^**d**^	11.76 ± 0.23^**d**^	11.75 ± 0.22^**d**^
	RBRW (%)	1.16 ± 0.09^**a**^	0.94 ± 0.04^**b**^	0.70 ± 0.03^**c**^	0.44 ± 0.01^**d**^	0.26 ± 0.01^**e**^	0.23 ± 0.01^**e**^	0.20 ± 0.00^**e**^
	% change	—	58.08	24.45	14.47	0.43	0	−0.09
	EQ	0.73 ± 0.05^**a**^	0.73 ± 0.03^**a**^	0.65 ± 0.03^**a**^	0.49 ± 0.02^**b**^	0.35 ± 0.01^**c**^	0.32 ± 0.01^**c**^	0.29 ± 0.001^**c**^
	OBW (g)	0.28 ± 0.02^**a**^	0.31 ± 0.02^**a**^	0.52 ± 0.03^**b**^	0.56 ± 0.04^**b**^	0.80 ± 0.03^**c**^	0.78 ± 0.05^**c**^	0.78 ± 0.03^**c**^
	CBW (g)	0.59 ± 0.01^**a**^	0.76 ± 0.02^**a**^	1.00 ± 0.03^**b**^	1.21 ± 0.06^**c**^	1.86 ± 0.06^**d**^	1.86 ± 0.05^**d**^	1.86 ± 0.06^**d**^

*Note:* The means with the different superscript letters show a significant difference (*p* < 0.05), but those with the same letters show no significant difference (*p* > 0.05) between ages.

Abbreviations: BRW, brain weight; BW, body weight; CBW, cerebellum weight; EQ: encephalization quotient; OBW: olfactory bulb weight; RBRW, relative brain weight.

**Table 2:** Volumetric parameters of the African grasscutter brain.

Volume		Number of samples (*n* = 42)						
		6 days	1 month	3 months	8 months	2 years	4 years	5 years
Average ± SEM (mL)	BRV (mL)	5.00 ± 0.29^**a**^	7.42 ± 0.20^**b**^	10.75 ± 0.17^**c**^	10.92 ± 0.27^**c**^	11.25 ± 0.21^**c**^	11.25 ± 0.21^**c**^	11.17 ± 0.28^**c**^
	OBV (mL)	0.33 ± 0.03^**a**^	0.37 ± 0.03^**a**^	0.60 ± 0.04^**b**^	0.90 ± 0.04^**c**^	0.95 ± 0.02^**c**^	0.95 ± 0.04^**c**^	0.95 ± 0.03^**c**^
	CBV (mL)	0.85 ± 0.06^**a**^	1.35 ± 0.06^**b**^	1.95 ± 0.04^**c**^	2.92 ± 0.06^**d**^	3.40 ± 0.06^**e**^	3.40 ± 0.07^**e**^	3.40 ± 0.06^**e**^

*Note:* The means with the different superscript letters show a significant difference (*p* < 0.05) in brain volume between ages. Those with the same letters show no significant difference (*p* > 0.05) between ages.

Abbreviations: BRV, brain volume; CBV, cerebellum volume; OBV, olfactory bulb volume.

## Data Availability

The data used to support the findings of this study are available from the corresponding author upon request.
